# Incidence of Periprocedural Myocardial Injury and Infarction After Intravascular Lithotripsy for Calcified Coronary Artery Stenosis

**DOI:** 10.1002/ccd.70868

**Published:** 2026-09-08

**Authors:** Gwaenaelle Aellen, Dorian Garin, Yannick Faucherre, Serban Puricel, Mario Togni, Stéphane Cook

**Affiliations:** ^1^ Cardiology, University and Hospital Fribourg Fribourg Switzerland

**Keywords:** calcified coronary lesions, coronary angiography, intravascular lithotripsy, lesion preparation, matched analysis, percutaneous coronary intervention myocardial injury, postprocedural myocardial infarction, real‐world study, stent expansion, troponin

## Abstract

**Background:**

Intravascular lithotripsy (IVL) has emerged as an effective technique for modifying heavily calcified coronary plaques, facilitating optimal stent expansion while minimizing procedural complications. However, data on periprocedural myocardial injury (PMI) and infarction (PPMI) following IVL remain scarce.

**Aims:**

To evaluate the incidence of PMI and PPMI after IVL in an unselected real‐world population undergoing percutaneous coronary intervention (PCI).

**Methods:**

All consecutive patients treated with IVL between May 2020 and December 2022 at a single tertiary center were included. Pre‐ and post‐procedural cardiac biomarkers (high‐sensitivity troponin T, CK, CK‐MB) and electrocardiograms were systematically analyzed. PMI and PPMI were defined according to the Fourth Universal Definition of Myocardial Infarction. A matched control cohort (2147 non‐IVL PCI patients) was used to compare biomarker dynamics.

**Results:**

Among 90 patients (mean age 73 ± 9 years; 77% male), diameter stenosis decreased from 55.2% ± 15.5% before IVL to 40.3% ± 15.7% immediately after IVL, with a final residual stenosis of 5.5% ± 18.5% after PCI and increased median minimal lumen diameter from 1.22 [0.72–1.72] mm to 3.04 [2.64–3.57] mm. High‐sensitivity troponin and CK‐MB levels rose modestly following IVL‐assisted PCI (*p* < 0.001), indicating mild myocardial injury. PMI was present in 49 patients (54%). Two patients met the criteria for PPMI, and no major procedural complication occurred. In an exploratory matched‐control analysis, IVL was associated with a moderate but statistically significant increase in biomarkers compared with matched controls (Cohen's d = 0.40).

**Conclusion:**

In this single‐center observational study, IVL was associated with limited myocardial injury, while clinically overt PPMI remained uncommon. These findings support the favorable safety profile of IVL as a plaque‐modification strategy for complex calcified coronary lesions.

**ClinicalTrials.gov Identifier:** NCT04185285.

**Abbreviations**
ACS: acute coronary syndromes
CABG: coronary artery bypass graft
CCS: chronic coronary syndromes
CI: confidence interval
CK: creatine kinase
DES: drug‐eluting stents
ECG: electrocardiogram
IVL: intravascular lithotripsy
MI: myocardial infarction
MLD: minimal lumen diameter
PCI: percutaneous coronary intervention
PMI: periprocedural myocardial injury
PPMI: periprocedural myocardial infarction (type 4a MI)

## 1 Introduction

Management of calcified coronary lesions remains one of the principal challenges in percutaneous coronary intervention (PCI). Heavy calcification hampers optimal stent delivery and expansion, thereby increasing the risk of procedural failure and periprocedural complications [1, 2, 3]. Conventional plaque‐preparation techniques—such as high‐pressure non‐compliant or cutting balloon dilatation, and rotational or orbital atherectomy—are effective but demand substantial operator expertise and carry an inherent risk of complications, including dissection, perforation, distal embolization, and no‐reflow phenomena [1, 3].

Recently, intravascular lithotripsy (IVL) has emerged as a promising alternative. This technique combines a low‐pressure balloon with an acoustic shockwave generator that fractures superficial and deep calcium while preserving surrounding soft tissue [1, 2, 3, 4]. In the pivotal Disrupt CAD III trial involving more than 400 patients, procedural success reached 92.4%, with 92.2% freedom from major adverse cardiovascular events at 30 days [1, 3]. A pooled analysis of Disrupt CAD I‐IV (628 patients) confirmed these findings, demonstrating the strong reproducibility and safety of IVL [5]. In real‐world settings, the BMC2 registry reported a rapid increase in IVL use, with outcomes comparable to those of atherectomy—both in terms of technical success and complication rates [6]. Moreover, the BENELUX registry highlighted that imaging‐guided procedures (IVUS/OCT) were as safe and effective as those performed under fluoroscopy alone, despite treating more complex lesions [7]. Meta‐analyses have reinforced these observations. A 2024 review including 38 studies and nearly 3000 patients reported a clinical success rate of 93% and a technical success rate of 97%, with very low rates of major complications [8]. However, none of these pivotal trials or registries systematically reported the incidence of distal embolization, periprocedural myocardial infarction (PPMI), or periprocedural myocardial injury (PMI), events that could theoretically result from the mobilization of calcific or thrombotic debris by shockwave energy.

To address this knowledge gap, we analyzed the first 100 patients treated with coronary IVL at our center. Our primary objective was to determine the prevalence of PMI and PPMI after IVL in an unselected patient population.

## 2 Methods

### 2.1 Study Population

The Cardio‐FR registry at University and Hospital Fribourg includes all patients aged ≥ 18 who are admitted for PCI and have provided written informed consent [9, 10, 11]. The study included an unselected real‐world population comprising both elective and acute coronary syndrome presentations, including CCS, unstable angina, NSTEMI, STEMI, and staged PCI procedures. The registry collects comprehensive data on baseline patient characteristics, procedural details, in‐hospital outcomes, and annual clinical follow‐ups conducted via regular telephone contacts. Stent allocation is done randomly using a pre‐established distribution key based on the day of the annual calendar. We included all patients who underwent PCI with IVL balloon (Shockwave IVL, Johnson & Johnson) between May 2020 and December 2022 and having completed the prospective 1‐year clinical follow‐up. The exclusion criteria were refusal or the inability to provide informed consent, absence of IVL use (*n* = 3), incomplete or missing information (*n* = 7), or loss of clinical follow‐up.

The Cardio‐FR registry complied with the Helsinki Declaration and was approved by the local ethics committee (003‐REP‐CER‐FR). The registry is registered in ClinicalTrials.gov, number NCT04185285.

### 2.2 PCI and Medications

Procedures were performed via the femoral or radial artery with a 6 French guiding catheter. Standard interventional techniques were used and performed according to practice guidelines. Use of intravascular imaging (OCT or IVUS) was at operator discretion. Pre‐procedural antithrombotic regimen was systematically achieved with aspirin (250–500 mg IV bolus for those not under treatment and 100 mg for those already under aspirin and then 100 mg/day for all) and unfractionated heparin (70 IU/kg), whereas cangrelor and tirofiban were administered at operator discretion. The coronary segments selected for IVL were determined by the operator based on angiographic characteristics and clinical judgment. Vessel preparation before IVL was performed using semi‐compliant, high‐pressure non‐compliant balloons, or cutting balloons at operator discretion.

IVL (Shockwave C2+, Johnson & Johnson, USA) was performed according to the manufacturer's standard protocol, with 60 pulses per treatment cycle. All patients received either a minimum 600 mg loading dose of clopidogrel, 180 mg of ticagrelor, or 60 mg prasugrel before or immediately after the procedure. Lifelong ≥ 100 mg daily aspirin and either 75 mg daily clopidogrel, 90 mg twice daily ticagrelor, or 10 mg prasugrel for a minimum of 3 months were prescribed. For patients on oral anticoagulation therapy, 100 mg of aspirin was given once daily for minimum 1 week, and clopidogrel 75 mg daily for 3–6 months in addition to oral anticoagulation. Other medications were prescribed as per standard of care. All patients were monitored for 4–12 h in an intermediate care unit and underwent systematic baseline cardiac biomarker assessment before PCI, followed by repeat measurements typically performed between 6 and 24 h after the procedure according to institutional practice. Additional measurements were obtained when clinically indicated. A standard 12‐lead electrocardiogram (ECG) was systematically recorded before PCI, immediately after the procedure, and concomitantly with post‐procedural biomarker assessments. Additional ECGs were performed when clinically indicated.

### 2.3 Data Collection and Endpoint Definition

For each case, pre‐ and post‐procedural laboratory values were collected, including high‐sensitivity cardiac troponins (hs‐Tn, T) and creatine kinase (CK) levels. Post‐IVL ECGs were analyzed individually. All analyses were performed in the central chemistry laboratory. All ECGs were analyzed independently by the internal review board and classified as abnormal when ischemic changes met the diagnostic thresholds defined by the European Society of Cardiology (ESC) guidelines. Post‐procedural echocardiography was not performed systematically but was obtained when clinically indicated, particularly in patients with suspected procedural complications, significant biomarker elevation, hemodynamic instability, or new ischemic ECG abnormalities.

PMI and periprocedural myocardial infarction (PPMI, type 4a MI) were defined according to the Fourth Universal Definition of Myocardial Infarction [12] and contemporary consensus recommendations regarding PMI and infarction [13, 14].

PMI was defined as an elevation of cardiac troponin values above the 99th percentile upper reference limit (URL) after PCI in patients with normal baseline values, or *a* > 20% increase in patients with elevated but stable or falling baseline troponin values.

PPMI (type 4a MI) was defined as a post‐procedural elevation of cardiac troponin values > 5 times the 99th percentile URL in patients with normal baseline values, or *a* > 20% rise from an elevated but stable or falling baseline value provided that at least one value exceeded 5 times the 99th percentile URL, together with at least one of the following: new ischemic ECG changes, angiographic evidence of flow‐limiting complications, development of pathological Q waves, or imaging evidence of new loss of viable myocardium.

In addition to biochemical and electrocardiographic analyses, detailed angiographic data were collected for all patients. Each coronary angiogram was independently reviewed by the investigator to assess the coronary segment treated with IVL.

### 2.4 Quantitative Coronary Angiography

Coronary angiograms were digitally recorded at baseline and immediately after the procedure and were assessed at the angiographic core laboratory of the University Fribourg (Head: GA). Quantitative coronary angiography was performed at three predefined procedural time points: before IVL, immediately after IVL balloon treatment, and at the completion of PCI after stent implantation. The angiographic projection that best visualized the stenosis was used for all analyses. Patients received nitroglycerine before angiography, and measurements were performed on cineangiograms. The contrast‐filled, nontapered tip of the catheter was used for calibration. Digital angiograms were analyzed with the use of an automated edge‐detection system (CAAS II, Pie Medical Imaging). Acute gain was defined as the difference between the final post‐procedural and pre‐procedural minimal lumen diameter (MLD). Binary restenosis was defined as > 50% diameter stenosis.

### 2.5 Exploratory Matched‐Control Analysis

All patients in the institutional “all‐comers” PCI registry who had paired pre‐ and post‐procedure high‐sensitivity cardiac troponin T measurements (*n* = 5636) were considered potential controls. Each IVL patient (index case) was matched with eligible control patients sharing the following predefined characteristics: sex, age (5‐year strata), clinical presentation (STEMI, NSTEMI, unstable angina, or chronic coronary syndrome), and baseline troponin concentration (±5 ng/L). No propensity score was calculated. Controls falling outside this calliper were discarded, and any index patient without at least one eligible match was removed. This matched comparison was intended as an exploratory analysis rather than a formal propensity score‐matched analysis.

### 2.6 Statistical Analysis

Categorical variables are presented as counts and percentages, whereas continuous variables are expressed as means and standard deviations or as medians with 25%–75% interquartile ranges, according to their distribution. Normality and homoscedasticity of continuous variables were assessed by visual inspection of histograms and Q−Q plots and further verified using the Shapiro−Wilk test. Categorical variables were compared using the chi‐square or Fisher's exact test, as appropriate. Continuous variables were analyzed using the Student's t‐test or the Mann−Whitney *U* test, according to their distribution. For paired data (e.g., pre‐ vs. post‐procedure comparisons), normality of paired differences was assessed using the Shapiro‐Wilk test. Normally distributed differences were analyzed with the paired *t*‐test or repeated‐measures ANOVA, while the Wilcoxon signed‐rank or Friedman test was used when normality assumptions were not met. Missing values were excluded from analysis. Paired analyses were performed using complete paired observations available for each biomarker. For the matched‐control analysis, the baseline balance on troponin was verified using the Mann−Whitney test. The increase in troponin was compared between groups using both the Welch *t*‐test (unequal variances) and the Mann−Whitney *U* test.

Effect size was expressed as Cohen's *d*, and nonparametric 95% confidence intervals (CIs) for differences in means and medians were obtained using a percentile bootstrap (10,000 resamples, sampling independently within each group).

All statistical analyses were performed using Python (Wilmington, USA) version 3.13.0 with dedicated packages: NumPy (1.24.3), pandas (2.0.1), psycopg2 (2.9.10), statsmodels (0.14.4), SciPy (1.15.2), matplotlib (3.10.1), and seaborn (0.13.2). The workflow was executed in Visual Studio Code (v1.95) connected to a local PostgreSQL database (v14.15) administered via DBeaver (v24.3.4). A two‐tailed significance level of *α* = 0.05 was applied to all statistical tests.

## 3 Results

### 3.1 Study Population

From an initial dataset of 100 patients, 10 were excluded due to predefined criteria: 3 because IVL was not performed, and 7 due to incomplete clinical or procedural data. The final study population therefore consisted of 90 patients (Figure 1). Baseline clinical characteristics are summarized in Table 1. The mean age was 73.2 ± 9.4 years, and 77% of patients were male. The most prevalent cardiovascular risk factors were hypertension (91%), Dyslipidaemia (86%), and diabetes mellitus (34.4%). Seventeen patients (18%) were active smokers, while six (6%) had recently quit smoking. Regarding cardiovascular history, 40% of patients had a previous myocardial infarction, 53% had undergone prior PCI, and 12% had a history of coronary artery bypass grafting (CABG). Compared with the general population from CardioFR (Table 2), this cohort was older and exhibited a higher prevalence of arterial hypertension, diabetes mellitus, and dyslipidaemia, reflecting the selection of patients with advanced coronary artery disease undergoing PCI.

**Figure 1:**
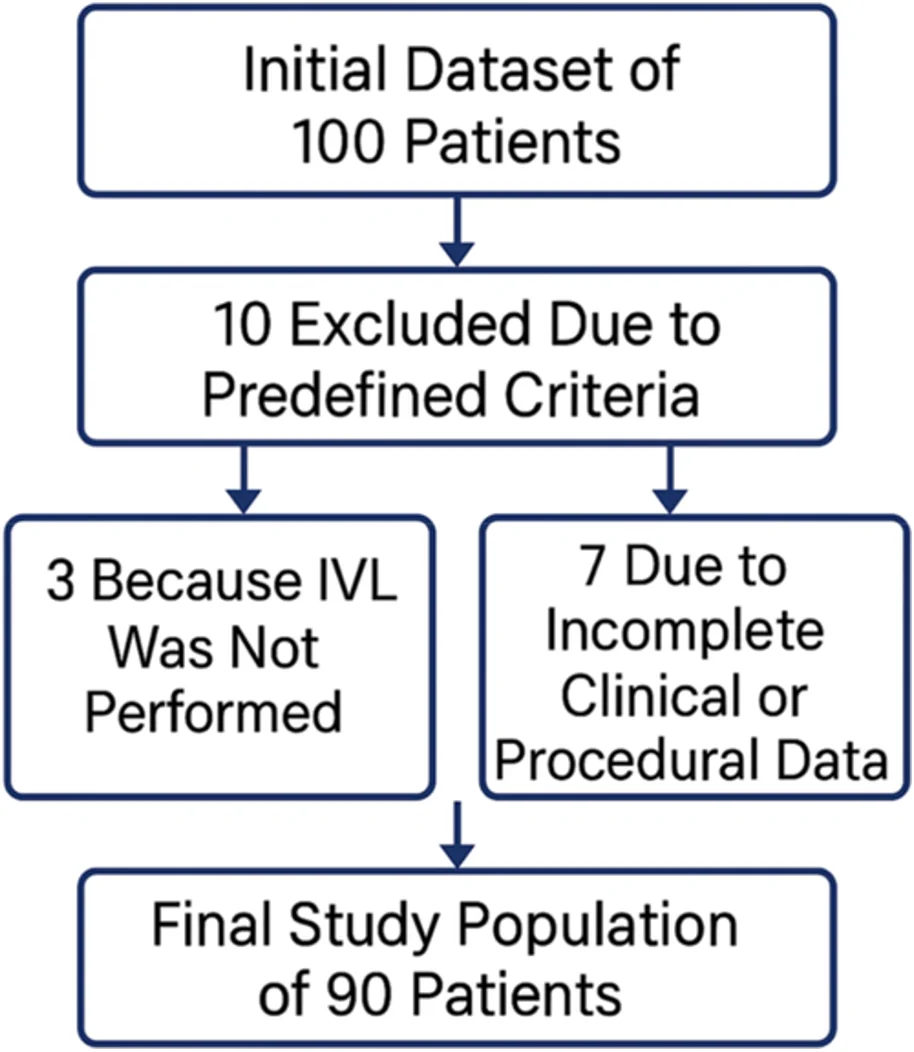
Study flow chart. Of the initial dataset comprising 100 patients, 10 were excluded according to predefined criteria. Three patients were excluded because IVL was not performed, and seven due to incomplete clinical or procedural data. The final study population, therefore, consisted of 90 patients. IVL, intravascular lithotripsy. [Color figure can be viewed at wileyonlinelibrary.com]

**Table 1:** Baseline patient‐, lesion‐ and procedural characteristics.

Patient characteristics	IVL cohort *N* = 90
Age, mean ± SD	73.2 ± 9.4
Male, *n* (%)	70 (77.7%)
Body mass index, mean ± SD	26.4 ± 4.8
Diabetes mellitus, *n* (%)	31 (34.4%)
‐Non‐insulin dependent, *n* (%)	21 (23.3%)
‐Insulin dependent, *n* (%)	10 (11.1%)
Dyslipidaemia, *n* (%)	78 (86.6%)
Arterial hypertension, *n* (%)	82 (91.1%)
Current smoker, *n* (%)	17 (18.8%)
Previous MI, *n* (%)	36 (40%)
Previous PCI, *n* (%)	48 (53.3%)
Previous CABG, *n* (%)	11 (12.2%)
ACS, STEMI *n* (%)	11 (12.2%)
ACS, NSTEMI *n* (%)	26 (28.9%)
CCS, *n* (%)	53 (58.9%)
Staged PCI, *n* (%)	14 (15.6%)
Number of vessels treated per patient, mean ± SD	1.1 ± 0.4
Number of lesions treated per patient, mean ± SD	1.6 ± 0.9
−1 lesion, *n* (%)	50 (55.6%)
−2 lesions, *n* (%)	32 (35.6%)
−3 lesions, *n* (%)	6 (6.7%)
−4 and more lesions, *n* (%)	2 (2.2%)
LVEF, % ± SD	52.0 ± 16.8
Lesion‐ and procedural characteristics	
Number of stents per patient, mean ± SD	1.47 ± 0.93
Maximum stent diameter per lesion, mm ± SD	3.21 ± 0.54
Total stent length per lesion, mm ± SD	36.57 ± 22.60
Maximal inflation pressure, atm ± SD	14.27 ± 3.24
Pre‐dilatation, *n* (%)	83 (92.2%)
Post dilatation, *n* (%)	65 (72.2%)
IVL, *n* (%)	100%
IVL diameter, mm ± SD	2.92 ± 0.44

*Note:* Values are presented as mean ± standard deviation or number (%).

Abbreviations: ACS, acute coronary syndromes; CABG, coronary artery bypass graft surgery; CCS, chronic coronary syndromes; IVL, intravascular lithotripsy; LVEF, left ventricular ejection fraction; MI, myocardial infarction; (N‐)STEMI, (Non‐)ST‐segment elevation myocardial infarction; PCI, percutaneous coronary intervention.

**Table 2:** Matched comparison IVL cohort versus control group.

	IVL cohort *N* = 90	Control group *N* = 2147	*p* value
Demographics			
Age, mean ± SD	73.2 ± 9.4	71.2 ± 6.3	0.37
Male, *n* (%)	70 (77.7%)	1593 (74.2%)	0.74
Body mass index, mean ± SD	26.4 ± 4.8	27.5 ± 4.7	0.04
Risk factors			
Diabetes mellitus, *n* (%)	31 (34.4%)	342 (15.9%)	< 0.001
Dyslipidaemia, *n* (%)	78 (86.6%)	1717 (79.9%)	0.20
Arterial hypertension, *n* (%)	82 (91.1%)	1825 (85.1%)	0.11
Current smoker, *n* (%)	17 (18.8%)	628 (29.3%)	0.02
Medical history			
Previous MI, *n* (%)	36 (40%)	270 (12.6%)	< 0.001
Previous PCI, *n* (%)	48 (53.3%)	398 (18.6%)	< 0.001
Previous CABG, *n* (%)	11 (12.2%)	146 (6.8%)	0.14
Clinical presentation			
Acute coronary syndromes, *n* (%)	39 (40.2%)	897 (41.7%)	0.70
Procedural characteristics			
Staged PCI, *n* (%)	14 (14.6%)	352 (16.4%)	0.71
Number of vessels treated, mean ± SD	1.1 ± 0.4	1.1 ± 0.3	0.90
Number of lesions treated, mean ± SD	1.6 ± 0.9	1.3 ± 0.6	0.001
LVEF, mean ± SD	52.0 ± 16.5	54.3 ± 13.1	0.14

*Note:* Values are presented as mean ± standard deviation or number (%).

Abbreviations: CABG, coronary artery bypass graft surgery; MI, myocardial infarction; PCI, percutaneous coronary intervention; LVEF, left ventricular ejection fraction.

### 3.2 Procedural Angiographic Measurements

Quantitative coronary angiography demonstrated a reduction in mean diameter stenosis from 55.2% ± 15.5% before IVL to 40.3% ± 15.7% immediately after IVL balloon treatment, reflecting the acute effect of calcium modification. Following stent implantation and procedural optimization, the final residual diameter stenosis further decreased to 5.5% ± 18.5% at the completion of PCI. The MLD rose from 1.22 [0.72–1.72] mm pre‐procedure to 3.04 [2.64–3.57] mm after IVL (*p* < 0.001), followed by a slight reduction to 2.54 [2.19–2.97] mm at the end of the procedure (*p* < 0.001) (Figure 2). The acute gain from baseline to the end of PCI, defined as the difference between final post‐PCI and baseline pre‐PCI MLD, was 1.44 ± 0.72 mm. When analyzing the entire procedure from baseline to the end of PCI, maximal lumen diameter increased significantly across pre‐, during‐, and post‐procedure measurements (*p* < 0.001, Friedman test). Similarly, MLD changed significantly from 1.15 ± 0.46 mm pre‐procedure to 3.11 ± 0.59 mm after IVL to 2.59 ± 0.60 mm at PCI end (*p* < 0.001, repeated‐measures ANOVA). (Table 3).

**Figure 2:**
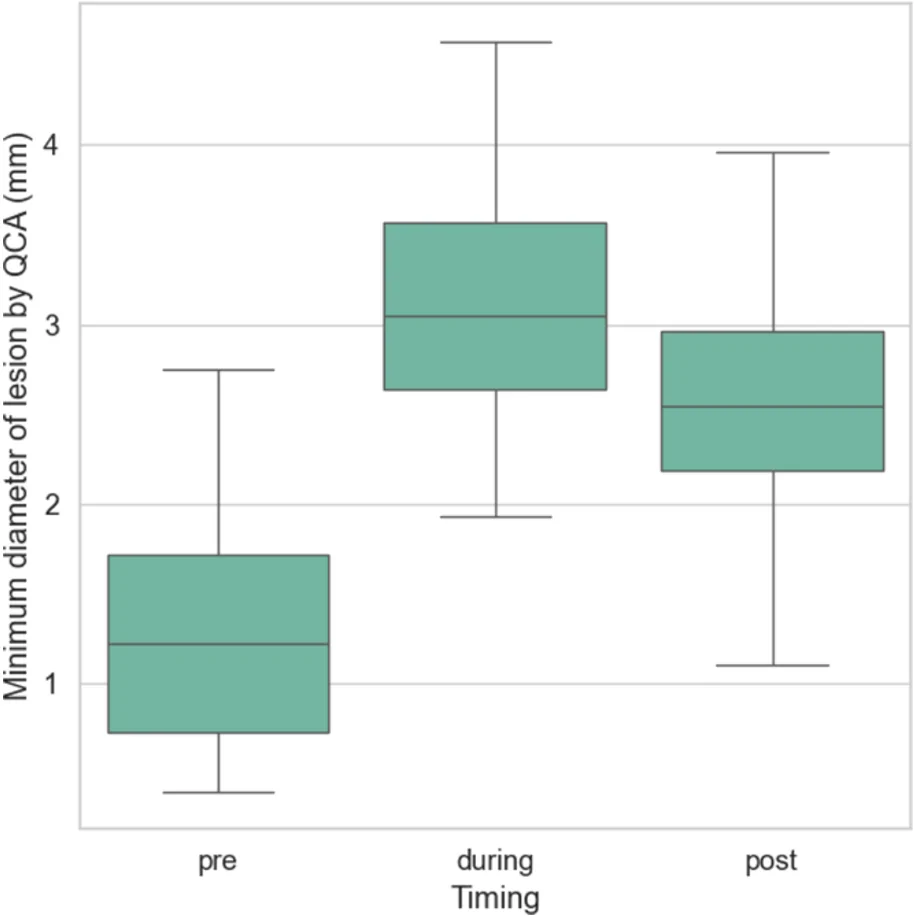
Distribution of the minimal lumen diameter (MLD) assessed by quantitative coronary angiography (QCA) before, during, and after percutaneous coronary intervention with IVL. IVL, intravascular lithotripsy; QCA, quantitative coronary angiography. [Color figure can be viewed at wileyonlinelibrary.com]

**Table 3:** Quantitative coronary angiography measurements.

	IVL cohort *N* = 90
Pre‐procedure	
MLD, mm ± SD	1.15 ± 0.46
RVD, mm ± SD	2.60 ± 0.63
Diameter stenosis, % ± SD	55.2 ± 15.5
After IVL	
MLD, in‐segment, mm ± SD	3.11 ± 0.59
End of PCI	
MLD, in‐segment, mm ± SD	2.59 ± 0.60
RVD, mm ± SD	2.78 ± 0.50
Diameter stenosis, % ± SD	5.53 ± 18.54
Acute gain, in‐segment, mm ± SD	1.44 ± 0.72

*Note:* Values are presented as mean ± SD. Corresponding median [IQR] values used for paired statistical comparisons are reported in the Results section. MLD, reference vessel diameter, and acute gain are reported in millimeters, whereas diameter stenosis is reported as percentage (%).

Abbreviations: IVL, intravascular lithotripsy; MLD, minimal lumen diameter; RVD, reference vessel diameter.

### 3.3 PMI and ‐Myocardial Infarction (PPMI)

Pre‐ and post‐procedure CK values did not differ significantly (142.9 ± 198.2 vs. 401.6 ± 1559.0 U/l; *p* = 0.237). In contrast, CK‐MB increased from 23.9 ± 17.6 U/l to 38.4 ± 139.8 U/l (*p* = 0.001) and high‐sensitivity troponin levels from 133.9 ± 367.4 to 838.6 ± 6271.0 (*p* < 0.001). Figure 3 illustrates the individual pre‐ and post‐procedural hs‐cTnT values. Troponin measurements were available in all patients for descriptive analyses, whereas complete paired measurements suitable for paired comparison were available in 76 patients. Although hs‐cTnT levels increased following IVL‐assisted PCI in most patients, the distribution was markedly heterogeneous, with the majority exhibiting modest biomarker elevations and only a limited number of patients demonstrating pronounced increases. This pattern is reflected by the moderate upward shift in the median hs‐cTnT value despite the presence of several high post‐procedural outliers. Consistently, PMI was present in 49 patients (54%; [95% CI, 48%–69%]) and 2 (2.2%) fulfilled the diagnosis of PPMI (Table 4).

**Figure 3:**
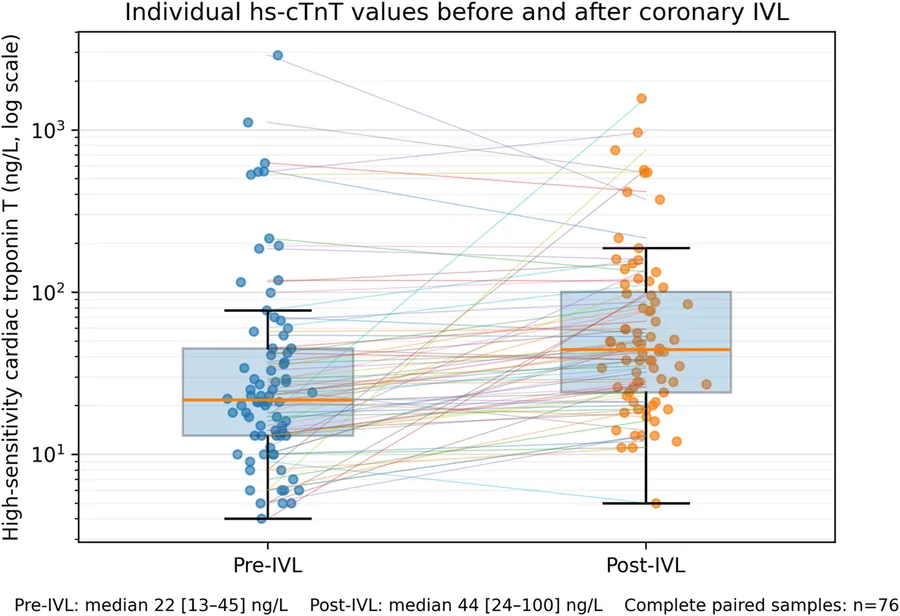
Individual pre‐ and post‐procedural high‐sensitivity cardiac troponin *T* values after coronary IVL. Each line represents one patient. Boxes indicate median and interquartile range. The y‐axis is displayed on a logarithmic scale. Only patients with complete paired pre‐ and post‐procedural hs‐cTnT measurements (*n* = 76) are displayed. IVL, intravascular lithotripsy; hs‐cTnT, high‐sensitivity cardiac troponin T. [Color figure can be viewed at wileyonlinelibrary.com]

**Table 4:** Cardiac biomarkers and electrocardiographic findings before and after percutaneous coronary intervention with intravascular lithotripsy (IVL).

	IVL cohort *N* = 90
Pre‐IVL	
Troponin ng/L ± SD	133.9 ± 367.4
CK U/L ± SD	142.9 ± 198.2
CK‐MB U/L ± SD	23.9 ± 17.6
Post‐IVL	
Troponin ng/L ± SD	838.6 ± 6271.0
CK, U/L ± SD	401.6 ± 1559.0
CK‐MB, U/L ± SD	38.4 ± 139.8
ECG modifications, *n* (%)	6 (7.6%)
Type 4a MI, *n* (%)	2 (2.2%)

*Note:* Values are presented as mean ± standard deviation or number (%). ECG modifications and the incidence of type 4a myocardial infarction are reported for the post‐procedural period.

Abbreviations: CK, creatine kinase; ECG, electrocardiogram; IVL, intravascular lithotripsy; MI, myocardial infarction.

### 3.4 Matched Control Analysis

Matching yielded 89/90 index patients and 2147/5636 controls whose baseline troponin concentrations were statistically indistinguishable (17 [IQR 17] ng/L vs. 15 [IQR 17] ng/L; *p* = 0.59). The median rise (Δ) was 11 ng/L [IQR 23] in the index group versus 2 ng/L [IQR 10] in controls, a median difference of +9 ng/L (bootstrap 95% CI +5 to +15; *p* < 0.0001). The mean rise (Δ) was 774 ± 6319 ng/L in the IVL group versus 117 ± 1161 ng/L in controls, giving a mean difference of +657 ng/L (bootstrap 95% CI 113–2131; *p* = 0.018).

The corresponding Cohen's *d* = 0.40, indicating a moderate effect size. Overall, IVL was associated with a moderate but statistically significant increase in troponin levels compared with matched controls.

## 4 Discussion

This study evaluated the incidence of PMI and PPMI following IVL for calcified coronary lesions. Three main findings emerged. First, device deliverability was high, allowing successful IVL delivery in 96.8% of cases (90/93 lesions). Second, quantitative coronary angiography demonstrated a pronounced mechanical effect of IVL, with a substantial acute luminal gain corresponding to a 170% increase in MLD, consistent with effective calcium modification and vessel expansion. Third, although biomarker‐defined myocardial injury was common after IVL‐assisted PCI, biomarker elevations were generally modest and clinically relevant PPMI remained rare. These findings suggest that biomarker elevation after PCI was generally limited and was only rarely associated with clinically overt type 4a myocardial infarction.

These findings are in line with prior evidence from pivotal trials and large real‐world data sets. The Disrupt CAD III trial demonstrated high procedural success and low rates of major adverse cardiovascular events, confirming the safety and effectiveness of IVL in severely calcified de novo coronary lesions under controlled trial conditions [1]. More recently, contemporary registry data from the BMC2 consortium showed that IVL use has rapidly expanded in routine practice and is associated with procedural success and in‐hospital adverse event rates comparable to atherectomy, even in older and more comorbid populations often excluded from pivotal trials [6]. In addition, a large multicenter systematic review and meta‐analysis encompassing nearly 3,000 patients confirmed high clinical and procedural success rates, significant acute luminal gain, and a low incidence of periprocedural complications across a broad spectrum of lesion and patient complexity [8].

Importantly, our real‐world cohort extends these observations by focusing on PMI, an aspect that has been only sparsely addressed in prior IVL studies. While modest biomarker release was observed, its magnitude was limited and not associated with clinical, electrocardiographic, or angiographic sequelae. Several mechanisms may account for this phenomenon. Acoustic pressure waves generated by IVL may mobilize microcalcific debris, transiently affecting microvascular perfusion, while balloon inflation itself inevitably induces short periods of ischemia in the treated segment. However, consistent with prior trial and registry data, these effects appear clinically negligible, reinforcing the concept that IVL achieves effective calcium modification with minimal myocardial injury.

The clinical significance of isolated post‐procedural biomarker elevations after complex PCI remains debated, particularly in the era of highly sensitive cardiac troponin assays. Although biomarker‐defined myocardial injury was relatively frequent in our cohort, clinically overt ischemic complications and type 4a myocardial infarction remained uncommon. These findings suggest that most biomarker elevations observed following IVL‐assisted PCI may reflect limited procedural myocardial injury rather than clinically significant infarction. Recent studies have emphasized that isolated post‐procedural biomarker elevations detected by highly sensitive troponin assays may not systematically reflect clinically meaningful myocardial infarction [15].

Overall, our findings complement existing evidence by suggesting that IVL achieves high angiographic efficacy while maintaining a favorable myocardial safety profile. Although supported by an exploratory matched‐control analysis, these observations should be interpreted cautiously given the observational design and the possibility of residual confounding.

## 5 Limitations

This study has several limitations. First, its single‐center design limits causal inference and generalizability. Second, documentation of the exact number of IVL pulses was incomplete for some procedures, which may have introduced minor variability. Finally, although a matched‐analysis was performed, residual confounding remains possible because matching did not account for several lesion‐specific and procedural variables known to influence biomarker release, including calcification severity, lesion complexity, total stent length, and number of treated lesions. Therefore, the matched comparison should be considered exploratory and hypothesis‐generating rather than a formal matched comparative analysis.

In addition, several clinically relevant differences persisted between the IVL and matched control groups despite matching, reflecting the higher complexity and cardiovascular risk profile of patients requiring IVL. These residual imbalances may have influenced biomarker release and further limit the interpretation of comparative analyses.

In addition, the absence of systematic mechanistic imaging and long‐term outcome correlations limits the interpretation of the clinical significance of post‐procedural biomarker elevations. Larger multicenter prospective studies integrating intracoronary imaging and long‐term clinical follow‐up are needed to better define the prognostic relevance of myocardial injury after IVL.

Despite these limitations, the study provides valuable real‐world data on the biochemical and procedural effects of IVL in an unselected patient cohort.

## 6 Conclusion

IVL safely achieves effective plaque modification and vessel expansion in calcified coronary lesions. Although biomarker‐defined myocardial injury was relatively frequent following IVL‐assisted PCI, clinically overt type 4a myocardial infarction remained uncommon. Overall, these findings support the favorable procedural safety profile of IVL in complex calcified coronary lesions.

## Conflicts of Interest

The authors declare no conflicts of interest.

## Data Availability

The data that support the findings of this study are available on request from the corresponding author. The data are not publicly available due to privacy or ethical restrictions.
